# The Relationship of T Helper-2 Pathway Components Interleukin-4, Interleukin-10, Immunoglobulin E, and Eosinophils with Prognostic Markers in Non-Hodgkin Lymphoma: A Case-Control Study

**DOI:** 10.4274/tjh.2013.0328

**Published:** 2014-12-05

**Authors:** Nil Güler, Engin Kelkitli, Hilmi Atay, Dilek Erdem, Hasan Alaçam, Yüksel Bek, Düzgün Özatlı, Mehmet Turgut, Levent Yıldız, İdris Yücel

**Affiliations:** 1 19 Mayıs University Faculty of Medicine, Department of Hematology, Samsun, Turkey; 2 19 Mayıs University Faculty of Medicine, Department of Oncology, Samsun, Turkey; 3 19 Mayıs University Faculty of Medicine, Department of Biochemistry, Samsun, Turkey; 4 19 Mayıs University Faculty of Medicine, Department of Biostatistics, Samsun, Turkey; 5 19 Mayıs University Faculty of Medicine, Department of Pathology, Samsun, Turkey

**Keywords:** chemokines, cytokines, Lymphocytes, non-Hodgkin lymphoma, Th2 pathway

## Abstract

**Objective:** Increased risk for non-Hodgkin lymphoma (NHL) is associated with infections and environmental agents. We hypothesized that these factors chronically trigger the T helper-2 (Th2) pathway and result in lymphoma. We investigated the role of the Th2 pathway by exploring the relationships between components of the Th2 pathway, interleukin (IL)-10, IL-4, immunoglobulin E (IgE), and eosinophils, and prognostic markers of NHL.

**Materials and Methods:** Thirty-one NHL patients and 27 healthy controls were enrolled. IL-10, IL-4, IgE, and eosinophils were measured. IL-4 and IL-10 were analyzed with the enzyme amplified sensitivity immunoassay method.

**Results:** High IL-10 levels were correlated with several poor prognostic features, short early survival, and lymphopenia. There was a positive correlation between albumin and IL-4 levels and a negative correlation between IL-10 and albumin. There was no relationship related with eosinophils and IgE. We found remnant increased IL-4, which could be a clue for the triggering of the Th2 pathway in the background.

**Conclusion:** There is a need for differently designed studies to detect the place of the Th2 pathway in NHL.

## Abstract (tr)

**Amaç:** Non-Hodgkin lenfoma (NHL) riski enfeksiyonlar ve çevresel ajanlarla artmış olarak ilişkilendirilmiştir. Hipotezimize göre bu faktörlere kronik olarak maruz kalmak T helper-2 (Th2) yolağını aktifler ve lenfomaya sebep olur. Bu amaçla Th2 yolağı komponentleri olan IL-10, IL-4, IgE, ve eozinofille, NHL’nin prognostik belirteçleri arasındaki ilişkiye baktık.

**Gereç ve Yöntemler:** Otuz bir NHL hastası ve 27 sağlıklı kontrolde IL-10, IL-4, IgE ve eozinofil değerlerine bakıldı. IL-4 ve IL-10 EASIA metodu ile ölçüldü.

**Bulgular:** Yüksek IL-10 düzeyleri pek çok kötü prognostik özellikle, çok kısa yaşam süresi ve lenfopeni ile ilişkiliydi. Albümin açısından IL-10 ile negatif ilişki, IL-4 ile pozitif ilişki bulundu. Eozinofil ve IgE ile ilgili herhangi bir ilişki kurulamadı. Bazı hastalarda gözlenen artmış IL-4 geri planda kalmış Th2 yolağı aktivasyonunun bir ip ucu olabilir.

**Sonuç:** Th2 yolağının NHL patogenezindeki yerini tespit etmek için farklı dizaynlarda çalışmalara ihtiyaç vardır.

## INTRODUCTION

Increased risk for Non-Hodgkin lymphoma (NHL) is associated with infections, environmental agents, and immune suppression. The T helper-2 (Th2) immune reaction is typically characterized by expression of interleukin (IL)-4, IL-5, IL-9, IL-10, and IL-13; the recruitment of eosinophils, basophils, and mast cells; and immunoglobulin (Ig) G-to-IgE antibody class switching [1]. Exposure to an antigen may cause an allergic reaction, but chronic exposure to the same allergen at a low dose can cause immune tolerance via T regulatory (T-reg) cell-associated Th2 suppression [2]. Anergy is defined as the inability of antigen-specific cells to generate an allergic reaction to an antigen [3].

IL-10 has a role in anergy as well as in immune suppression, whereas IL-4 plays a predominant role in allergy [3]. IL-10 is produced mainly by Th2 cells, T-reg cells, monocytes, and B lymphocytes and, in small amounts, by Th1 cells. IL-10 inhibits the proliferation of Th1 and Th2 cells in response to specific antigens. IL-10 also inhibits the production of gamma-interferon (IFN-γ) and IL-2 by Th1 cells; the production of IL-4 and IL-5 by Th2 cells; the production of IL-6, IL-8, IL-12, TNF-α, and IL-1β by mononuclear phagocytes; and the production of TNF-α and IFN-γ by natural killer cells. It further inhibits monocytes [3,4,5]. Therefore, IL-10 is known as a strongly inhibitory cytokine. IL-10 causes differentiation of T-reg cells from T helper cells. T-reg cells secrete IL-10, IFN-γ, TGF-β, and IL-5 [3]. T-reg cells also suppress functions of Th1 and Th2 cells [6]. Secretion of IL-4 by the Th2 pathway induces allergy. Desensitization treatments can be done if an allergic patient is chronically treated with a related allergen at a low dose. Allergic individuals develop anergy via IL-10.

**Figure 1:**
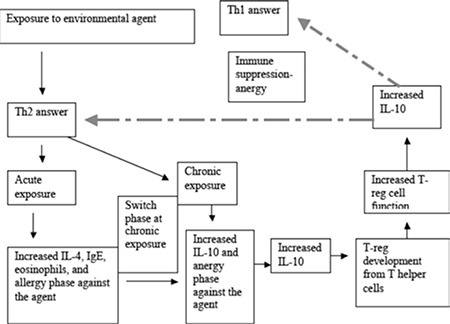
Presentation of the hypothesis. Dashed arrows indicate suppressive effect.

We hypothesized that chronic stimulation by environmental agents triggers the Th2 pathway and anergy. The Th2 pathway is shifted from IL-4 to IL-10 in anergy. It is well known that high IL-10 triggers the differentiation of T-reg cells from T helper cells. Once T-reg cells are increased, these cells will start to inhibit functions of Th2 and Th1 cells and start to secrete their own IL-10. In addition, if high levels of IL-10 persist, it causes serious immune suppression. Therefore, we examined the relationships between prognostic markers in NHL and components of the Th2 pathway, such as IL-10, IL-4, IgE, and eosinophils. Figure 1 illustrates our hypothesis.

## MATERIALS AND METHODS

Thirty-one newly diagnosed NHL patients and 27 healthy controls were enrolled. The median ages of patients and controls were 64 (range: 19-85) and 26 (range: 23-60) years, respectively. All participants were free of history of allergy, dermatitis, or parasites in stool samples, because allergies and parasites can trigger the Th2 pathway and could cause misleading results.

Twenty-seven patients had diffuse large B cell lymphoma (DLBCL) and 4 patients had T-cell lymphoma. The characteristics of the patients are given in Table 1.

Initial blood samples were collected to test IL-4, IL-10, and IgE levels. Patient pretreatment values for eosinophils, hemoglobin, lymphocytes, lactate dehydrogenase (LDH), C-reactive protein (CRP), sedimentation, b2 microglobulin, and albumin were collected from the patient records. A second blood sample for IL-4 and IL-10 was collected from only the patients after completion of the 4 cycles of chemotherapy. The chemotherapy regimens were decided by their physicians. The blood samples for IL-10 and IL-4 were collected into tubes with no anticoagulant. The samples were centrifuged within 2 h at 4000 rpm for 10 min, and the serum was separated and kept at -80 °C.

The IL-4 and IL-10 concentrations were analyzed from serum samples using the enzyme amplified sensitivity immunoassay (EASIA) method in accordance with the instructions of the kit’s manufacturer (DIAsource ImmunoAssays, Cat. No. KAP1281 and KAP1321, Belgium). These IL-4 and IL-10 assays are specific for endogenous human IL-4 and IL-10, respectively. The samples were processed in duplicate. The detection limits of the kits for IL-4 and IL-10 are 1.2 pg/mL and 1.6 pg/mL, respectively. The intraassay and interassay variation of the IL-4 kit was 3.8% and 4.5%, respectively. The intraassay and interassay variation of the IL-10 kit was 2.8% and 2.8%, respectively. The results are presented in pg/mL.

The Eastern Cooperative Oncology Group (ECOG) performance status system and the Ann Arbor staging system were used. International Prognostic Index (IPI) scores were categorized as low risk (scores 0-2) or high risk (scores 3-5).

T-cell lymphoma patients were treated with CHOP (cyclophosphamide, doxorubicin, vincristine, and prednisone) chemotherapy. DLBCL patients were treated with rituximab plus CHOP.

Overall survival (OS) was determined as the time between the diagnosis of lymphoma and the last evaluation in the hospital or death for any reason during the study.

The university’s local ethics committee approved this study and all participants gave informed consent. This study is in accordance with the Helsinki Declaration of 1975.

**Statistical Analysis**

Statistical analyses were performed using SPSS 15.0. The normality of distribution was checked by Kolmogorov-Smirnov test. For variables not confirming to normal distribution comparisons were analyzed with the Mann-Whitney U-test. The Wilcoxon signed-rank test, Kruskal-Wallis test, Spearman test, Pearson chi-square test, and Fisher exact test were also used. Statistical significance was accepted at p<0.05. The Kaplan-Meier test and log rank analysis were performed for survival analysis.

## RESULTS

IL-4 values were a median of 19.41 (min: 18.67, max: 43.05) and a mean of 20.72±4.8 in the control group. They were a median of 19.41 (min: 19.04, max: 2081.7) and a mean of 96.27±369.65 in the patient group.

IL-10 values were a median of 7.1 (min: 6.6, max: 8.24) and a mean of 7.2±0.5 in the control group. They were a median of 8.2 (min: 6.1, max: 3155.4) and a mean of 163.93±574.5 in the patient group.

The maximum values of IL-10 and IL-4 in the control group were used as cut-off values for determining increased IL-10 and IL-4 levels. Thus, the cut-off values were 8.24 pg/mL and 43.05 pg/mL for IL-10 and IL-4, respectively. As mentioned above, the medians of IL-10 and IL-4 in the control group were 7.1 pg/mL and 19.4 pg/mL, respectively. Fifteen of the 31 lymphoma patients (48%) exhibited increased IL-10 and 5 patients (16%) exhibited increased IL-4 levels. Four patients exhibited elevations of both IL-4 and IL-10.

There was a positive correlation between IL-4 levels and albumin (r=0.44, p=0.013), whereas a negative correlation was observed between IL-10 and albumin (r=-0.46, p=0.01) using Spearman’s correlation test. IL-10 was positively correlated with CRP (r=0.58, p=0.001) and LDH (r=0.764, p<0.001). 

The pretreatment IL-10 values were significantly correlated with IPI [low and high: median of 6.65 (range: 6.12-28) and 29.13 (range: 6.65-3155), respectively; p=0.001], with lymphoid tissue size [≤5 and >5 cm: median of 7.18 (range: 6.12-687) and 43.06 (range: 6.65-3155), respectively; p=0.016], with ECOG performance [<2 and ≥2: median of 7.18 (range: 6.12-28) and 61.01 (range: 6.65-3155), respectively; p=0.007), and with stage (early and advanced; p=0.003).

Because of these correlations, we hypothesized that high IL-10 levels negatively affected prognosis, given their relationship with high LDH, high CRP, high IPI score, lymphoid tissue size of greater than 5 cm, poor ECOG performance, advanced stage, and albumin. By contrast, we hypothesized that high IL-4 levels positively affected prognosis because of their positive relationship with albumin in our study. To test this hypothesis, we classified the patients according to IL-10 and IL-4 values. We thought if IL-10 is a poor prognostic marker and IL-4 is a good prognostic marker, the group with the best prognostic profile for interleukins would have low IL-10 and high IL-4, and the group with the worst prognostik profile for interleukins would have high IL-10 and low IL-4. A group with relatively good prognostic profile for interleukins would have low IL-10 and low IL-4; a group with relatively poor prognostic profile for interleukins would have high IL-10 and high IL-4.

Groups were established as follows: group 1, IL-10 of ≤8.24 and IL-4 of >43.05 (1 patient); group 2, IL-10 of ≤8.24 and IL-4 of ≤43.05 (15 patients); group 3, IL-10 of >8.24 and IL-4 of >43.05 (4 patients); group 4, IL-10 of >8.24 and IL-4 of ≤43.05 (11 patients). Because it only included a single patient, group 1 was not evaluated. We did not observe a significant difference between groups 2 and 3 with respect to albumin (p=0.548), while there was a difference between groups 2 and 4 (p=0.005) and between groups 3 and 4 (p=0.026). The significant difference between groups 3 and 4 was noteworthy because both of these groups consisted of patients with high IL-10. However, IL-4 was higher in group 3 and lower in group 4. The highest albumin values were observed in group 3. As mentioned above, there was a negative correlation between IL-10 levels and albumin in the entire patient sample (r=-0.46, p=0.01). Despite the high IL-10 level, albumin levels were high in group 3.

**Figure 2:**
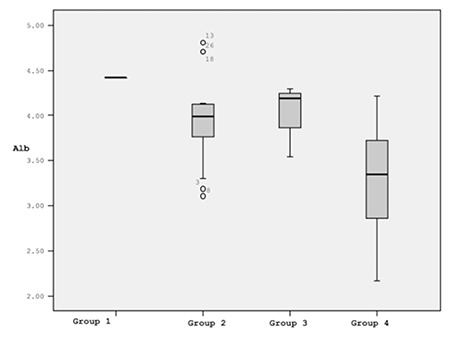
Mean albumin values (g/dL) after removal of group 1 (with 1 patient only) from analysis.

The lack of a difference between group 2 and group 3 was also important. IL-10 levels were low in group 2. The albumin values in group 3 did not differ from those of group 2 despite high IL-10 levels. These results suggest that IL-4 alleviates or restores the repressive effects of IL-10 on albumin (Figure 2).

Seven of 15 patients with high IL-10 (46%) and 4 of 16 patients with low IL-10 (25%) died during the follow-up for a total number of 11 deceased patients. Ten of these 11 patients died before the completion of the fourth cycle of chemotherapy. Our mean observation period was 281.44 days (range: 19-402). The minimum observation time in living patients was 112 days. Therefore, we relied on short-time surveying.

The highest IL-10 value in living patients was 107.1 pg/mL, 13-fold higher than the cut-off value. This value was found in patient number 21. This patient also exhibited a very high IL-4 value of 2081 pg/mL, 48-fold higher than the cut-off value.

For survival analysis, the patients were classified into 3 groups according to the cut-off value (8.24 pg/mL) and the highest value (107 pg/mL) in living patients: Group A, IL-10 of ≤8.24 (16 patients); group B, 8.24 < IL-10 ≤107 (11 patients); and group C, IL-10 of >107 (4 patients). The Kaplan-Meier test and log rank analysis were performed for the survival analysis.

**Figure 3:**
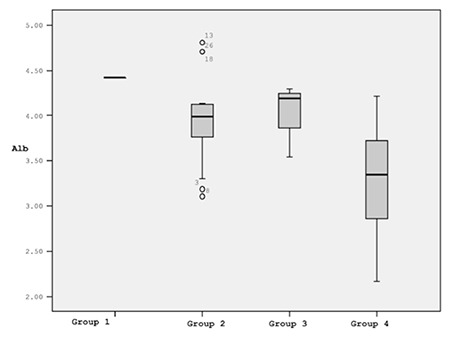
Survival analysis as days related to IL-10 levels.

The mean survival times were 323 days in group A, 285 days in group B, and 38.7 days in group C. The differences were statistically significant (p=0<0.001). Severe early survival differences were noted in group C (Figure 3). Groups A, B, and C contained 15 and 1, 9 and 2, and 3 and 1 DLBCL and T-cell lymphoma patients, respectively.

IgE levels were high in 8 patients. There was no relationship between increased IgE levels and age, IPI score, LDH, ECOG performance, stage, IL-10, IL-4, albumin, or lymphocyte count. There were only 2 patients with high eosinophil count.

The pretreatment lymphocyte count was ≤1x109/L in 11 (35.5%) patients. Seven (47%) of 15 patients with high IL-10 and 4 (25%) of 16 patients with low IL-10 had lymphocyte counts of ≤1x109/L. We observed a difference between the participants with low IL-10 (≤8.24), consisting of 27 controls and 16 patients (43 individuals total), and the participants with high IL-10 (>8.24; 15 patients). Lymphocyte counts were decreased in the latter group (p=0.002).

Differences in the relationships between IL-4 and age, CRP, hemoglobin, extranodal involvement, ECOG performance, stage, lymphopenia, LDH, lymphoid tissue size, IPI score, ≥2 microglobulin, or B symptoms were not observed.

The characteristics of patients with T-cell lymphoma are shown in Table 2.

## DISCUSSION

In this study, high IL-10 levels were correlated with several poor prognostic features, including low albumin. However, IL-4 was positively correlated with albumin. In addition, IL-4 was able to overcome the negative effects of IL-10 on albumin. To our knowledge, this is the first study to detect a positive relationship between IL-4 and albumin and the ability of IL-4 to overcome the effects of IL-10 on albumin in NHL patients. 

IL-4 is an agent used in experimental treatment for NHL. The rationalization for this treatment is based on the observation of the inhibitory effect of IL-4 on NHL B cells and cancer cells in vitro [7,8].

In the literature, IL-10 is generally connected to the poor prognostic factors of NHL. One of the most noteworthy relevant studies was performed by Blay et al. They observed detectable IL-10 levels in 46% of patients with active disease. Detectable IL-10 levels were related to very short survival [9]. However, Cortes et al. could not observe a relationship with complete remission, failure-free survival, or OS. There was also no relationship between IL-10 and any prognostic factors except B symptoms [10].

Lech-Maranda et al. observed a relationship between detectable IL-10 levels and age of >60, ECOG status of ≥2, advanced stage, bulky tumor mass, high LDH, high IPI score, ≥2 microglobulin, anemia, existence of B symptoms, low albumin, low CR rate, and shorter progression-free survival and OS [11]. Nacinovic-Duletic et al. reported similar results. The patients with high IL-10 exhibited shorter survival [12]. However, Guney et al. observed only the relationship between IL-10 and high LDH and bone marrow involvement [13]. They detected significant decreases in IL-10 levels after chemotherapy. Fabre-Guillevin et al. did not find any relationship between IL-4 or IL-10 and complete remission, failure-free survival, or OS [14].

As mentioned before, Lech-Maranda et al. observed a negative correlation between albumin and IL-10 [11]. Our finding was consistent with this result; in addition, we observed a positive effect of IL-4 on albumin.

Lymphopenia is related to poor prognosis in many cancers [15,16]. In our study, the pretreatment lymphocyte count was negatively correlated with IL-10. To our knowledge, this is the first study in which lymphopenia was found to be related to high IL-10 levels in NHL. Some researchers have observed this relationship in sepsis [17,18].

We cannot define the source of high IL-10 levels in our study; it could be Th2, T-reg, or tumor cells. According to our hypothesis, NHL pathogenesis starts with Th2 pathway activation. We know that the increased secretion of IL-10 by Th2 cells can promote the development of T-reg cells from T helper cells, and newly developed T-reg cells begin to secrete their own IL-10 and inhibit both Th1 and Th2 functions [3,6]. As mentioned above, IL-10 is a strong inhibitor cytokine. In situations of very highly increased IL-10 levels, the inhibitor effect of IL-10 will be particularly enormous and will result in strong inhibition of Th1 and Th2 cell function. With the suppressive effect of IL-10, Th2 cannot produce IL-4. Our interpretation of the increase in IL-4 that was observed in some patients (5 patients with increased pretreatment IL-4 and 3 with increased posttreatment IL-4 values) is that there may be remnant Th2 pathway activation, which could have escaped the suppressive effect of IL-10. It is hard to show the initial activity of Th2 cells at the time of disease development because we are most likely catching patients after the switching of Th2 cell activity to increased T-reg cell activity and at a point when the disease is well established.

To our knowledge, this is the first study to investigate the place of the Th2 pathway in NHL through the components of Th2 by detection of IgE, eosinophils, IL-10, and IL-4.

IL-10 was related to very early death in this study. Therefore, we think that other treatment options may be more effective for patients with very high IL-10, such as anti-IL-10 antibody in addition to CHOP. However, one could also make a different comment: if an increasing IL-10 level is accompanying NHL, the increased IL-10 may be a reaction of the immune system, and supporting this reaction may help to control the aggression of lymphoma.

**Acknowledgment**

Special thanks to Timuçin Güler for interpretation of our results.

**Conflict of Interest Statement**

The authors of this paper have no conflicts of interest, including specific financial interests, relationships, and/or affiliations relevant to the subject matter or materials included.

## Figures and Tables

**Table 1:**
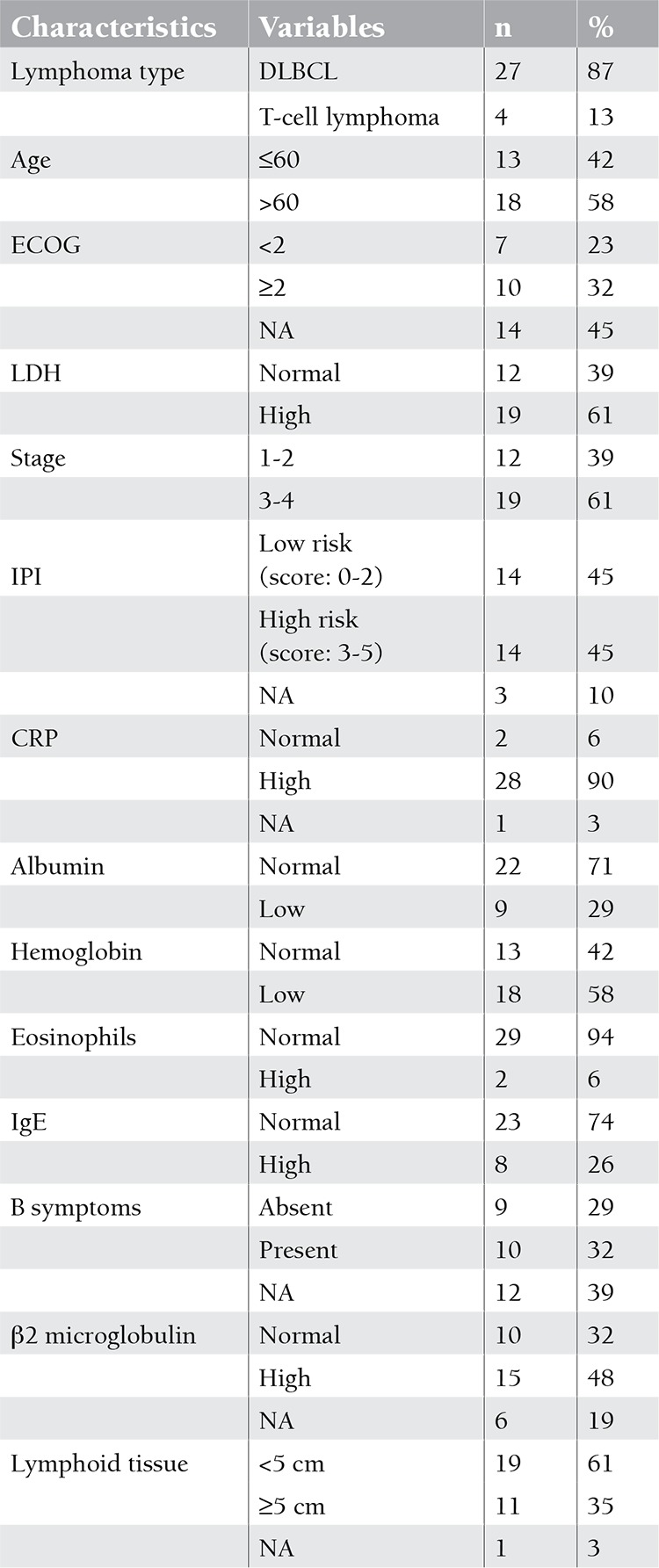
The characteristics of the patients.

**Table 2:**
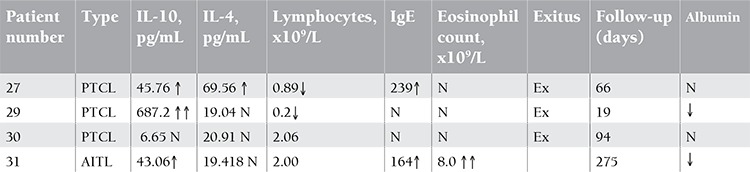
The characteristic of patients with T-cell lymphoma.
